# Ecological selection pressures for C_4_ photosynthesis in the grasses

**DOI:** 10.1098/rspb.2008.1762

**Published:** 2009-02-25

**Authors:** Colin P. Osborne, Robert P. Freckleton

**Affiliations:** Department of Animal and Plant Sciences, University of SheffieldSheffield S10 2TN, UK

**Keywords:** C_4_ photosynthesis, adaptation, water-use efficiency, aridity, shade, phylogeny

## Abstract

Grasses using the C_4_ photosynthetic pathway dominate grasslands and savannahs of warm regions, and account for half of the species in this ecologically and economically important plant family. The C_4_ pathway increases the potential for high rates of photosynthesis, particularly at high irradiance, and raises water-use efficiency compared with the C_3_ type. It is therefore classically viewed as an adaptation to open, arid conditions. Here, we test this adaptive hypothesis using the comparative method, analysing habitat data for 117 genera of grasses, representing 15 C_4_ lineages. The evidence from our three complementary analyses is consistent with the hypothesis that evolutionary selection for C_4_ photosynthesis requires open environments, but we find an equal likelihood of C_4_ evolutionary origins in mesic, arid and saline habitats. However, once the pathway has arisen, evolutionary transitions into arid habitats occur at higher rates in C_4_ than C_3_ clades. Extant C_4_ genera therefore occupy a wider range of drier habitats than their C_3_ counterparts because the C_4_ pathway represents a pre-adaptation to arid conditions. Our analyses warn against evolutionary inferences based solely upon the high occurrence of extant C_4_ species in dry habitats, and provide a novel interpretation of this classic ecological association.

## 1. Introduction

The majority of terrestrial plant species use the C_3_ photosynthetic pathway. However, the efficiency of this process is compromised by photorespiration, and its rate is strongly limited by CO_2_ diffusion from the atmosphere. Photorespiration increases at low CO_2_ concentrations and high temperatures, and CO_2_ limitation is accentuated by the reduction of stomatal aperture under arid conditions (Björkman 1971; Osmond *et al.* 1982). The evolution of C_4_ photosynthesis has solved each of these problems via a suite of physiological and anatomical adaptations that concentrate CO_2_ at the site of carbon fixation, minimize photorespiration and raise the affinity of photosynthesis for CO_2_ at low mesophyll concentrations (Björkman 1971; Osmond *et al.* 1982). As a consequence, C_4_ plants have the potential to achieve higher rates of photosynthesis than their C_3_ counterparts, particularly at high irradiance (Black *et al.* 1969). Since C_4_ photosynthesis draws mesophyll CO_2_ down to lower concentrations than the C_3_ type, it also allows stomatal conductance to be reduced, leading to greater water-use efficiency than the C_3_ pathway under the same environmental conditions (Downes 1969). The C_4_ pathway is therefore classically viewed as an adaptation to declining levels of atmospheric CO_2_ (Ehleringer *et al.* 1991), and hot, open, arid environments (Björkman 1971; Loomis *et al.* 1971).

Approximately half of the world's grass species use C_4_ photosynthesis (Sage *et al.* 1999*a*), and these plants dominate grassland and savannah ecosystems in warm climate regions (Sage *et al.* 1999*b*). They also include economically important food crops such as maize and sugarcane, and biofuel crops such as switchgrass and *Miscanthus*. Recent phylogenetic data suggest that the C_4_ pathway evolved in 9–18 independent clades of grasses during the past 32 million years (Myr) (Christin *et al.* 2008; Vicentini *et al.* 2008). However, only the earliest of these evolutionary origins coincided with the major decline in CO_2_ that occurred during the Oligocene (32–25 Myr ago; Pagani *et al.* 2005; Christin *et al.* 2008; Roalson 2008; Vicentini *et al.* 2008). One phylogenetic analysis suggests that the evolution of the C_4_ pathway became more likely after the CO_2_ decrease (Christin *et al.* 2008), and complementary studies suggest that the C_4_ origination events were clustered in time (Vicentini *et al.* 2008), and occurred in grass clades that were already adapted to warm climates (Edwards & Still 2008). However, adaptive hypotheses about the suite of local ecological factors that are selected for C_4_ photosynthesis remain largely untested (Roalson 2008). Chief among these are the hypothesized roles of water deficits caused by aridity or salinity, and the formation of open habitats via disturbance (Sage 2001).

The C_4_ photosynthetic pathway offers grasses the potential to achieve higher rates of leaf carbon fixation with a similar or lower expenditure of water than C_3_ species (Loomis *et al.* 1971; Gifford 1974). It also maximizes dry matter production when water is available in limited pulses (Williams *et al.* 1998), and allows the conservation of water in a drying soil (Kalapos *et al.* 1996). These physiological benefits are moderated by a trade-off between the photosynthetic rate and the intrinsic water-use efficiency of C_4_ leaves (Meinzer 2003). However, they are consistent with the common occurrence of C_4_ grass species in seasonally arid ecosystems, deserts and on saline soils (Sage *et al.* 1999*b*). Compelling evidence for the ecological sorting of C_4_ species into drier habitats than C_3_ species was provided by a recent comparative study of the largely exotic Hawaiian grass flora (Edwards & Still 2008).

Despite their prevalence in dry habitats, C_4_ grasses also occupy a diverse range of mesic, shaded and flooded ecological niches, and the primary importance of aridity for the ecological success of these species has therefore been challenged (Ehleringer *et al.* 1997; Sage *et al.* 1999*b*; Keeley & Rundel 2003). Large-scale spatial patterns also highlight a more complex relationship with climate than predicted by water-use efficiency alone, with the biomass of C_4_ grasses relative to other plant functional types increasing, rather than decreasing, with rainfall across the Great Plains of North America (Paruelo & Lauenroth 1996). In fact, the potential for C_4_ photosynthesis to drive high rates of productivity means that there are sound theoretical reasons to expect a selective advantage for the pathway in moist soil environments, whenever high temperatures are coupled with moderate-to-high light availability (Long 1999; Sage *et al.* 1999*b*; Keeley & Rundel 2003; Sage 2004).

Spatial correlations with environmental variables suggest that some of the observed variation in the ecological niche of C_4_ grasses may be explained by the contrasts in the tolerance of aridity between different phylogenetic groups (Hartley 1950; Taub 2000). Unravelling the confounding effects of physiology and phylogeny will therefore be crucial if we are to make realistic predictions about the future impacts of increasing aridity on community composition in subtropical grasslands (Christensen *et al.* 2007), and move towards a greater understanding of the role of palaeoclimate change in driving the expansion of C_4_ grassland ecosystems in the geological past (Osborne 2008).

The aim of this study is to investigate the ecological selection pressures for C_4_ photosynthesis in the grasses, using the comparative method to test the alternative hypotheses of adaptation (Harvey & Pagel 1991). Drawing upon a recently published phylogeny (Christin *et al.* 2008), we have compiled a global habitat dataset for 117 genera of grasses, sampling each of the major clades and 15 independent C_4_ lineages. Analyses of these data address two key questions. First, which ecological factors have selected for the C_4_ pathway, in particular, is it an adaptation to aridity? And secondly, to what extent is variation in the ecological niches of different C_4_ plant groups explained by phylogenetic history? Our results are consistent with the hypothesis that selection for C_4_ photosynthesis occurred in open habitats but was independent of water availability, whereas subsequent evolutionary transitions into arid habitats were faster in C_4_ than C_3_ clades.

## 2. Material and methods

### (a) Phylogenetic framework

Phylogenetic relationships were based on the calibrated consensus tree of Christin *et al.* (2008). Species sampling for this tree was designed to include all postulated origins of the C_4_ photosynthetic pathway within the grasses, and to minimize the distance between the stem group and crown group nodes. The topology was obtained by Bayesian inference using the chloroplast DNA markers *rbcL* and *ndhF*, and calibrated using Bayesian molecular dating, with minimum ages for six nodes based on fossil evidence (Christin *et al.* 2008). Branch lengths are therefore proportional to time elapsed. The grass phylogeny was kindly provided by Pascal-Antoine Christin (University of Lausanne).

Since the complete phylogenetic analysis spanned the entire order Poales, we first extracted the 187 species belonging to the grass family Poaceae. The tree indicated that a number of genera were polyphyletic (e.g. *Panicum*, *Merxmuellera*), and these were removed as it was not possible to generate unequivocal trait data for these. One genus that appeared to be paraphyletic (*Brachiaria*) was combined together with its sister (*Urochloa*) to form a monophyletic clade. This procedure resulted in a phylogeny of 129 grass genera.

### (b) Ecological data

The photosynthetic type (C_3_ or C_4_) within each genus was assigned following Sage *et al.* (1999*a*). However, a number of genera could not be categorically assigned a photosynthetic type, since they contained C_3_, C_4_ and C_3_–C_4_ intermediate species (*Neurachne*, *Alloteropsis* and *Steinchisma*). Rather than excluding these genera from the analysis, we assigned photosynthetic type based on the majority of species (*Neurachne* and *Alloteropsis*=C_4_ and *Steinchisma*=C_3_), and tested the sensitivity of our analyses to this assumption by examining the effects of a reversal in the photosynthetic type for these genera.

Habitat and diversity data were then derived from the information compiled by Watson & Dallwitz (1992 onwards). For each genus, we recorded the number of species and type of habitats occupied, including information on water requirements (e.g. hydrophyte, xerophyte), tolerance of saline conditions (halophyte and glycophyte) and the occupation of shaded habitats (shaded and open). Water requirements were assigned a numerical score, giving equal weighting to the extremes (hydrophyte=5, helophyte=4, mesophyte=3 and xerophyte=1), and resulting in a continuous sequence of values for each genus. The habitat types occupied by each genus were then characterized using the mean and range of these values. Two further binary traits recorded the presence or absence of shade species, and the presence or absence of xerophytes. Since halophytes tolerate physiological drought imposed via high osmotic pressure, we also included genera containing halophytes in the ‘xerophyte’ category. However, all of the halophytic genera except one (*Spartina*) contained xerophytes. Habitat data were not available for all clades, and our final dataset included a total of 117 genera, sampling 15 out of the 17 hypothesized origins of C_4_ photosynthesis in the grasses (Christin *et al.* 2008). The full dataset is provided in table S1 in the electronic supplementary material.

### (c) Phylogenetic comparative analysis

In the first set of analyses we aimed to determine whether photosynthetic pathway is associated with several continuous ecological traits. Photosynthetic pathway was coded as a binary categorical variable (C_3_ versus C_4_). The number of species within a genus, and the mean and range of genus water requirements were coded as continuous variables. To test whether these were correlated with photosynthetic pathway, we used a generalized linear model in which the continuous variable was the dependent variable and the photosynthetic pathway a categorical predictor. In order to control for phylogenetic dependence we simultaneously estimated Pagel's *λ* (Pagel 1999) using the approach described in Freckleton *et al.* (2002). This parameter measures, and controls for, the degree to which the residual variation shows phylogenetic non-independence according to the predictions of a simple Brownian model of trait evolution. According to this, a value of *λ*=0 indicates that there is no phylogenetic dependence in the data, while *λ*=1 indicates that the residuals show strong phylogenetic dependence.

### (d) Modelling evolutionary pathways

In the second set of analyses, our objective was to model the transitions between C_3_ and C_4_ photosynthetic pathways and to determine whether these are associated with transitions between habitat types, specifically shaded versus open habitats, and xeric versus mesic ones. We modelled the evolutionary transitions using approaches described in Pagel (1994, 1999) and Pagel & Meade (2006). In brief, this method is based on a continuous-time Markov model, which models the transitions of discrete characters between states. For a pair of binary traits, there are four possible states (state 1=00, state 2=01, state 3=10, state 4=11) and eight parameters, which are the instantaneous rates of change between the states (denoted by *q*_*ij*_, measuring the rate of change from state *i* to *j*), assuming that instantaneously only a single change in one character may occur. The model was fitted using the reversible jump Markov chain Monte Carlo methods described in Pagel & Meade (2006) using the package BayesTraits (http://www.evolution.rdg.ac.uk/BayesTraits.html), and parameters were sampled from their posterior distributions.

In the first analysis, we wished to test whether transitions between C_3_ and C_4_ pathways were dependent on habitat openness. Thus, each genus was coded as either exclusively confined to open habitats (0) or sometimes/always occupying shaded habitats (1), and as being C_3_ (0) or C_4_ (1). We fitted the full model allowing for all single-step transitions between the states. In order to test the hypotheses concerning the rates of evolution between the states, we conducted three comparisons: firstly, we asked whether the rate of transition between C_3_ and C_4_ differed between open and shaded habitats (by contrasting rates *q*_13_ and *q*_24_). Secondly, we asked whether the rate of transition from open to shaded habitats differed between C_3_ and C_4_ lineages (by contrasting *q*_12_ and *q*_34_). And finally, we asked whether the transition from shaded to open habitats differed between C_3_ and C_4_ lineages (by contrasting *q*_21_ and *q*_43_).

In the second analysis, we tested whether the transitions between C_3_ and C_4_ pathways were accompanied by changes in the aridity of occupied habitat. Each genus was coded as being either exclusively confined to waterlogged/mesic habitats (0) or sometimes/always occupying xeric/saline habitats (1), and again we fitted a full model including eight parameters. From the posterior distribution of parameter estimates, we compared the distributions of the estimates of rates of transition from C_3_ to C_4_ in xeric and mesic habitats. Again, we used the fitted parameters to test three hypotheses: firstly, we asked whether the rate of transition between C_3_ and C_4_ pathways differed in mesic and xeric habitats (by contrasting *q*_13_ and *q*_24_). Secondly, we asked whether the rate of transition from mesic to xeric habitats differed between C_3_ and C_4_ lineages (by contrasting *q*_12_ and *q*_34_). And finally, we asked whether the transition from xeric to mesic habitats differed between C_3_ and C_4_ lineages (by contrasting *q*_21_ and *q*_43_).

To contrast *q*_*ij*_ and *q*_*kl*_, for each model in the posterior distribution we calculated the difference *q*_*ij*_−*q*_*kl*_. For the whole set of models in the posterior distribution, we then examined the distribution of values of these differences to determine whether there were systematic deviations from zero. These differences are presented in the supplementary information together with the estimated parameters for all models (see table S2 in the electronic supplementary material).

The possibility of evolutionary reversals from the C_4_ pathway to the C_3_ type remains a key area of uncertainty in phylogenetic models. Phylogenetic analyses of the numerous C_3_ and C_4_ clades in the subfamily Panicoideae suggest that the hypotheses of multiple evolutionary origins and/or reversions are equally parsimonious (Giussani *et al.* 2001) and, in the genus *Alloteropsis*, a C_4_ to C_3_ reversal is the single most parsimonious interpretation (Ibrahim *et al.* 2009). Although the convergent evolution of amino acid sequences in a C_4_-specific enzyme does provide compelling evidence for multiple C_4_ origins in this grass subfamily (Christin *et al.* 2007), phylogenetic analyses still indicate a high likelihood of reversion events in the Panicoideae (Vicentini *et al.* 2008).

However, one issue of concern in such analysis is that, when analysing the evolution of a binary trait, if one of the trait states has a higher speciation rate, reconstructions can appear to support the enhanced rates of reversals from rare to common states (Maddison 2006), and this problem affects the method used here. We note below that we find evidence consistent with higher rates of diversification in C_4_ grass clades, raising the possibility of a non-random distribution of extinction probabilities across C_3_ and C_4_ lineages.

Clearly, the issue of reversible transitions between photosynthetic pathways is contentious and must be considered in ecological models of C_4_ grass evolution. We therefore conducted two sets of analysis to consider the sensitivity of our results to this. In the first instance, we conducted the analysis as described above, including the possibility of reversions. We then re-analysed the data, prohibiting reversals from C_4_ to C_3_. This constrained model included six rather than eight parameters. We asked two further questions using the full, eight-parameter models; if they are possible, do C_4_ to C_3_ reversals depend on shading or aridity (*q*_31_ versus *q*_42_)?

## 3. Results

### (a) Comparative analysis

**Figure 1:**
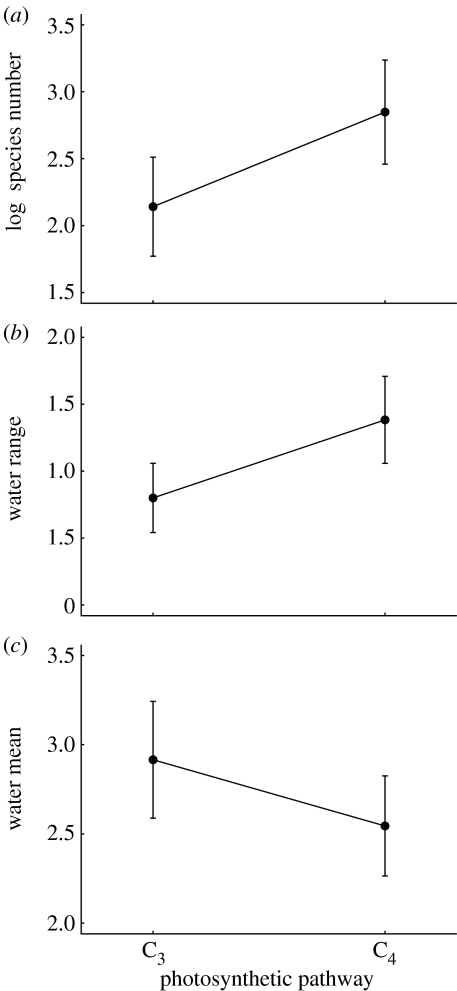
Species number and habitat water requirements in extant C_3_ and C_4_ genera. The plots show mean ±95% C.I. for (*a*) species number, (*b*) range of water requirements tolerated and (*c*) mean water requirements for each photosynthetic type.

Species number is significantly higher within C_4_ than C_3_ genera (table 1; figure 1*a*), and the range of habitat water requirements within each genus is significantly greater for the C_4_ than the C_3_ type (table 1; figure 1*b*). Species number is 33 per cent greater in C_4_ compared with C_3_ genera (figure 1*a*), while the range of habitat water requirements almost doubles (increasing by 85%; figure 1*b*). Neither shows significant phylogenetic dependence (*λ*=0; table 1). However, there is a significant linear association between species number and the range of habitat water requirements (*F*_1,90_=26.32, *p*=1.7×10^−6^). The range of habitats occupied within each genus explains about a quarter of its species number (*R*^2^=0.22). Critically, the introduction of photosynthetic type as a categorical predictor does not significantly improve the fit of this statistical model to the data (*F*_2,90_=1.88, *p*=0.17). This means that the observed association between species number and photosynthetic type may be entirely due to habitat diversity rather than a direct effect of C_4_ photosynthesis *per se*. In other words, C_4_ genera occupy a wider range of habitats and this, in turn, is associated with a larger number of species per genus.

The mean habitat water requirement is significantly lower in C_4_ than C_3_ genera (table 1; figure 1*c*), and shows a strong, statistically significant phylogenetic dependence (*λ*→1; table 1). Therefore, C_4_ genera occupy a wider range of drier habitats than their C_3_ counterparts, but different clades of grasses differ markedly in their habitat water requirements. These results are insensitive to the assumptions made about photosynthetic pathway in the genera *Neurachne*, *Alloteropsis* and *Steinchisma*.

### (b) Evolutionary transitions

**Figure 2:**
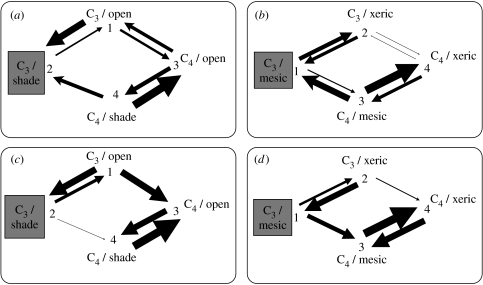
Models of the coevolution of photosynthetic pathway and habitat preference. Reversals from C_4_ to C_3_ photosynthesis are allowed in models (*a*,*b*), but prohibited in (*c*,*d*). Models (*a*,*c*) show preference for habitat openness, and (*b*,*d*) tolerance of habitat aridity. Grey-shaded boxes indicate the most likely ancestral condition, and arrow size is proportional to the rate/likelihood of transitions between character states.

Figure 2 summarizes the rates of evolutionary transitions between states, considering the phylogenetic tree as a whole, and all of the postulated origins of C_4_ photosynthesis. The rate estimates are summarized in table S2 in the electronic supplementary material, together with the credible intervals based on the distribution of rate estimates in the posterior. All of these results are insensitive to the assumptions made about photosynthetic pathway in the genera *Neurachne*, *Alloteropsis* and *Steinchisma*.

Evolutionary transitions from C_3_ to C_4_ photosynthesis are significantly faster in grass clades confined to open habitats (i.e. *q*_13_>*q*_24_; figure 2*a*,*c*), and this result is robust to assumptions about the possibility of reversions from C_4_ to C_3_ photosynthesis (figure 2*a* versus figure 2*c*). The same analysis shows that grass clades occupying shaded habitats are significantly more likely to become confined to open habitats if they are C_4_ than C_3_ (i.e. *q*_43_>*q*_21_; figure 2*a*,*c*). However, the rate of evolutionary transitions from open to shaded habitats is independent of photosynthetic type, and C_3_ and C_4_ species are therefore equally likely to adapt to shade (i.e. *q*_12_=*q*_34_; figure 2*a*,*c*). Again, these results are robust to the assumptions made about C_4_ to C_3_ reversions (figure 2*a* versus figure 2*c*). If C_4_ to C_3_ reversals are possible, they occur at the same rate (are equally likely) in open and shaded habitats (i.e. *q*_31_=*q*_42_; figure 2*a*).

**Figure 3:**
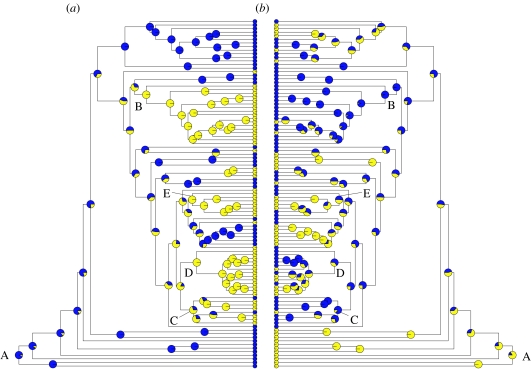
Likelihood of alternative ancestral states for nodes in the phylogenetic tree, showing (*a*) photosynthetic pathway (yellow circles, C_4_; blue circles, C_3_) and (*b*) preference for habitat openness (yellow circles, shade; blue circles, open habitat). See figure S1 in the electronic supplementary material for key to genera. Ancestral values were computed for individual traits using the likelihood method of Pagel (1994) and phylogenies drawn using the ace and plot.phylo functions in APE (Paradis *et al.* 2004).

**Figure 4:**
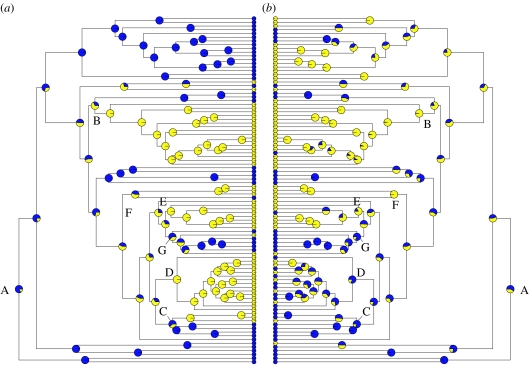
Likelihood of alternative ancestral states for nodes in the phylogenetic tree, showing (*a*) photosynthetic pathway (yellow circles, C_4_; blue circles, C_3_) and (*b*) preference for habitat aridity (yellow circles, xeric; blue circles, mesic). See figure S1 in the electronic supplementary material for key to genera. Ancestral values were computed for individual traits using the likelihood method of Pagel (1994) and phylogenies drawn using the ace and plot.phylo functions in APE (Paradis *et al.* 2004).

The likelihoods of ancestral character states at each node in the phylogeny are shown in figure 3, with a key to genera provided in figure S1 in the electronic supplementary material. The model indicates with a high posterior probability that the last common ancestor of the Poaceae was a C_3_ shade species (figure 3, node A). It also illustrates the most likely evolutionary pathway to C_4_ photosynthesis, whereby a transition into open habitats was a necessary pre-condition for the origin of the C_4_ pathway. For example, the model shows with high likelihood that the last common ancestors of the C_4_ clades Chloridoideae (figure 4, node B) and *x*=10 Paniceae (figure 4, node C) were confined to open habitats. However, the open habitat reconstructions for last common ancestors of the C_4_ clades Andropogoneae (figure 4, node D) and the ‘main clade’ of *x*=9 Paniceae (figure 4, node E) have lower associated probabilities.

Unexpectedly, evolutionary transitions from C_3_ to C_4_ photosynthesis occur at the same rate (are equally likely) in grass clades that contain xerophytic or halophytic species, and those confined to mesic or waterlogged habitats (i.e. *q*_13_=*q*_24_; figure 3*b*,*d*). However, the rate/likelihood of evolutionary transitions from mesic to xeric habitats is significantly higher in C_4_ than in C_3_ grass clades (i.e. *q*_34_>*q*_12_; figure 3*b*,*d*). By contrast, species are equally likely to become confined to mesic or waterlogged habitats if they are C_3_ or C_4_ (i.e. the rate of evolutionary transition from xeric to mesic habitats is independent of photosynthetic type, *q*_21_=*q*_43_; figure 3*b*,*d*). As in the previous analysis, these results are robust to the assumptions made about the possibility of C_4_ to C_3_ reversions (figure 3*b* versus figure 3*d*). If C_4_ to C_3_ reversals are possible, they depend significantly on habitat water availability, and evolutionary reversion is significantly faster/more likely in mesic or waterlogged habitats than xeric ones (i.e. *q*_31_>*q*_42_; figure 2*b*).

The second model of ancestral character states is shown in figure 4 (key to genera in figure S1 in the electronic supplementary material), and indicates that the most likely common ancestor of the Poaceae was a C_3_ species confined to mesic habitats (node A). It also illustrates important contrasts between clades in the habitat where the C_4_ pathway evolved. For example, the model shows with a high probability (greater than 80%) that the last common ancestors of the C_4_ clades Chloridoideae (figure 4, node B), ‘Arundinelleae’ (figure 4, node F) and the main clade of *x*=9 Paniceae (figure 4, node E) occupied xeric habitats, whereas ancestors of the Andropogoneae (figure 4, node D), the *x*=9 Paniceae clade containing *Echinochloa* and *Alloteropsis* (figure 4, node G) and *x*=10 Paniceae (figure 4, node C) were more likely confined to mesic habitats (probability greater than 80%). This contrast in the ancestral state of independent C_4_ clades illustrates how the phylogenetic correlation in mean habitat water requirements may arise (table 1).

## 4. Discussion

### (a) Ecological selection

Our three complementary analyses provide robust statistical support for the following adaptive hypothesis of C_4_ pathway evolution in the grasses. Selection for C_4_ photosynthesis occurs in open habitats, but may take place in mesic, arid or saline conditions. Once the pathway has evolved, C_4_ lineages adapt to arid and saline habitats at a faster rate than C_3_ lineages, and are more likely to become confined to open environments; C_4_ photosynthesis in the grasses therefore represents a pre-adaptation (exaptation) to xeric conditions. However, evolutionary transitions into shaded and mesic habitats are independent of photosynthetic type. If reversals from the C_4_ to C_3_ type occur, they do so in mesic or waterlogged habitats, irrespective of the habitat light regime. The net result of these evolutionary processes is that extant C_4_ genera occupy a drier range of habitats than their C_3_ counterparts. This association of photosynthetic pathway with aridity in extant genera may interact with temperature, but we were unable to test this with our dataset.

Seasonal aridity, fire, the activity of large mammalian herbivores and edaphic factors increase the availability of open habitats through the reduction of woody plant cover (Sankaran *et al.* 2008). Our data are therefore consistent with the hypothesis that these factors raise the likelihood of C_4_ pathway evolution in the grasses (Sage 2001). The strong statistical dependence of C_4_ pathway evolution on habitat openness is also consistent with the environmental responses of photosynthesis in extant C_3_ and C_4_ grasses: temperature and irradiance are greater in open than shaded environments, especially in the period after a disturbance event (Knapp 1984), which enhances the advantage of C_4_ photosynthesis for CO_2_ fixation over the C_3_ type (Black *et al.* 1969; Björkman 1971). Our finding that shade adaptation is independent of photosynthetic type is therefore surprising, especially since C_4_ grasses are virtually absent from the deep shade of forest floor environments (Sage 2001). However, the shade beneath trees in tropical woodlands and savannahs is associated with high soil moisture and nutrient contents, and the tolerance of low irradiance gives grasses the opportunity to exploit these soil resource patches (Ludwig *et al.* 2001).

The analysis of evolutionary transitions across the whole grass phylogeny provides no statistical evidence for an overall dependence of C_4_ pathway evolution on aridity. However, it does not exclude the possibilities that (i) arid or saline conditions may select for C_4_ photosynthesis in some grass clades (e.g. Chloridoideae) and not others (e.g. Andropogoneae) or (ii) high evaporative demand and soil drying between episodic rainfall events (Williams *et al.* 1998) or after fire (Knapp 1984) may be important selection pressures for C_4_ photosynthesis in mesic habitats. A previous comparative analysis suggested that the C_4_ pathway has evolved in grass clades of warm climate regions (Edwards & Still 2008), where high rates of evaporation and shallow rooting systems may lead to leaf water deficits of −1.5 MPa, even when the soil is wet (Le Roux & Bariac 1998). Although these adaptive interpretations are possible, they are not necessary, because our finding that C_4_ photosynthesis is a pre-adaptation to arid conditions is strongly supported across the whole phylogenetic tree. It is consistent with the well-known association between photosynthetic pathway and leaf water consumption (e.g. Black *et al.* 1969; Downes 1969). However, it warns against adaptive inferences based solely upon correlations in extant species between photosynthetic pathway and habitat aridity, such as those observed in our data (table 1) and by previous authors (Edwards & Still 2008).

### (b) Diversity and data quality

The association between species number and the range of habitats occupied by each genus could arise for a number of reasons. First, the origin of C_4_ photosynthesis may represent a ‘key innovation’ (Hunter 1998) that stimulates evolutionary diversification by increasing the rate of transition into xeric niches compared with the C_3_ type. In this case, ecological selection is implicated in both the origins of C_4_ photosynthesis and subsequent diversification within C_4_ grass clades. However, it is important to note that, while the number of species and range of habitats may on average be larger within each C_4_ than C_3_ genus, this does not mean that C_4_ grasses occupy a wider range of habitats overall. A second possible explanation for the observed correlation is sampling bias. If the sample of C_4_ grasses is biased towards large genera, then the wider habitat range could be a statistical artefact arising from the greater probability of encountering species from different habitats in large samples. Testing these alternative explanations will require phylogenetic measures of diversification rates, rather than the genus-based approach used here. This is because different genera may have begun to diverge at different times, and genus size depends crucially on the attention paid to each group by taxonomists.

The habitat data used in our analysis are simple, qualitative characterizations of the ecology of each genus. However, despite the basic nature of this information, we still found strong associations between photosynthetic pathway and habitat, with highly significant statistical support. The qualitative agreement between the three different analyses lends further confidence to our findings. While it is possible that the phylogeny may have biased sampling via the selection of species whose phylogenetic position is important, but whose ecology is atypical, this should have been counteracted by the explicit consideration of branch lengths in our analysis. A final sampling issue arises from our use of binary habitat traits, which potentially underestimate habitat diversity in large genera. However, the strong positive correlation between the range of water requirements and species number in each genus suggests that this did not bias our findings.

Our analysis suggested that the distribution of traits is consistent with the possibility of reversions from C_4_ to C_3_ types. This echoes the findings in other analyses (Ibrahim *et al.* 2009; Vicentini *et al.* 2008); however, we should be cautious about this conclusion at this stage. As noted previously, if we analyse traits that shape the phylogeny via speciation (or extinction) rates, then the outcome of the analyses can be misleading. The problem described by Maddison (2006) would arise in our dataset if the rate of speciation were greater in species with one photosynthetic pathway than the other, and the result in figure 1*a* indicates that this may have been the case, subject to the caveats above.

## 5. Conclusions

We have sought statistical evidence for an adaptive hypothesis of C_4_ pathway evolution in the grasses. Our analyses are consistent with the hypothesis that selection for C_4_ photosynthesis requires open environments, but indicate that the high occurrence of C_4_ clades in dry habitats arises because the pathway is a pre-adaptation to xeric conditions. These results provide a novel interpretation of the classic association of C_4_ plants with arid environments.

## Figures and Tables

**Table 1:** Results of generalized linear models testing for an association between photosynthetic pathway (C_3_ or C_4_) and species number or habitat characteristics. (‘Species number’ indicates the total number of species within each genus. ‘Water range’ and ‘water mean’ refer to the range and mean of habitat water categories, taken across all of the species within each genus. The results show the *F*-ratio, degrees of freedom (d.f.) and significance level (*p*-value) for photosynthetic pathway as a categorical predictor in each model. Pagel's *λ* estimates the degree of phylogenetic dependence in the data, ranging from 0 (no dependence) to 1 (strong dependence).)

variable	*F*-ratio	d.f.	*p*-value	*λ*
species number	6.95	1, 115	9.5×10^−3^	0.00
water range	7.78	1, 90	6.4×10^−3^	0.00
water mean	6.76	1, 90	1.1×10^−2^	0.83

## Supplementary Material

Table S1 Full dataset used in the analysis

Table S2 Fitted models

Figure S1 Key to the genera in figures 3 and 4
