# Supplementary material for: Ecological selection pressures for C4 photosynthesis in the grasses
Source: Proc Biol Sci. 2009 Feb 25;276(1663):1753–60. doi: 10.1098/rspb.2008.1762 (PMC2674487; doi:10.1098/rspb.2008.1762)
Supplement: Table S1 — Full dataset used in the analysis [file rspb20081762s02.doc]

**Table S1.** Genera and habitat data used in the analysis: Photosyn = photosynthetic type (0 = C_3_, 1 = C_4_); Species Number = number of species in the genus; Shade = occupation of shade habitats (0 = genus confined to open habitats, 1 = one or more species occur in shaded habitats); Water Mean = mean habitat water requirement (see text for details); Water Range = range of habitat water requirements (see text for details); Xero = occupation of xeric habitats (0 = glycophytic genus confined to mesic or waterlogged habitats, 1 = one or more species are xerophytic or halophytic). Also shown (right) are the raw habitat data upon which these scores are based (Watson & Dallwitz, 1992 onwards). Each ‘X’ records the presence of a species within the genus occupying a particular habitat type.

|  | | | **Derived habitat scores** | | | | **Raw habitat data** | | | | | | |
| --- | --- | --- | --- | --- | --- | --- | --- | --- | --- | --- | --- | --- | --- |
| **Genus** | **Photosyn** | **Species Number** | **Shade** | **Water Mean** | **Water Range** | **Xero** | **open** | **shade** | **5 hydrophyte** | **4 helophyte** | **3 mesophyte** | **1 xerophyte** | **halophyte** |
| *Mesosetum* | 1 | 35 | 0 |  |  |  | X |  |  |  |  |  |  |
| *Tatianyx* | 1 | 1 | 0 |  |  |  | X |  |  |  |  |  |  |
| *Homolepis* | 0 | 4 | 1 | 3.5 | 1 | 0 | X | X |  | X | X |  |  |
| *Oplismenopsis* | 0 | 1 |  | 4.5 | 1 | 0 |  |  | X | X |  |  |  |
| *Leptocoryphium* | 1 | 1 | 0 |  |  |  | X |  |  |  |  |  |  |
| *Hymenachne* | 0 | 5 |  | 4.5 | 1 | 0 |  |  | X | X |  |  |  |
| *Otachyrium* | 0 | 7 |  | 3.5 | 1 | 0 |  |  |  | X | X |  |  |
| *Steinchisma* | 0 | 4 |  | 3 | 0 |  |  |  |  |  | X |  |  |
| *Echinolaena* | 0 | 6 | 0 | 3.5 | 1 |  | X |  |  | X | X |  |  |
| *Axonopus* | 1 | 114 | 0 | 3.5 | 1 | 0 | X |  |  | X | X |  |  |
| *Ophiochloa* | 1 | 1 |  | 5 | 0 | 0 |  |  | X |  |  |  |  |
| *Anthaenantiopsis* | 1 | 4 | 0 |  |  |  | X |  |  |  |  |  |  |
| *Paspalum* | 1 | 320 | 0 | 3 | 3 | 1 | X |  |  | X | X | X | X |
| *Arundinella* | 1 | 55 | 0 | 3.5 | 1 | 0 | X |  |  | X | X |  |  |
| *Zea* | 1 | 1 |  | 3 | 0 | 0 |  |  |  |  | X |  |  |
| *Vetiveria* | 1 | 10 |  | 4 | 0 | 0 |  |  |  | X |  |  |  |
| *Bothriochloa* | 1 | 35 | 0 | 3 | 0 | 0 | X |  |  |  | X |  |  |
| *Capillipedium* | 1 | 14 | 0 |  |  |  | X |  |  |  |  |  |  |
| *Cymbopogon* | 1 | 40 | 0 | 2 | 2 | 1 | X |  |  |  | X | X |  |
| *Andropogon* | 1 | 100 |  | 2 | 2 | 1 |  |  |  |  | X | X |  |
| *Hyparrhenia* | 1 | 55 | 0 | 2 | 2 | 1 | X |  |  |  | X | X |  |
| *Coix* | 1 | 5 | 1 | 3.5 | 1 | 0 | X | X |  | X | X |  |  |
| *Microstegium* | 1 | 15 | 1 | 3 | 0 | 0 |  | X |  |  | X |  |  |
| *Eulalia* | 1 | 30 | 0 | 3.5 | 1 | 1 | X |  |  | X | X |  | X |
| *Arthraxon* | 1 | 7 | 1 | 3.5 | 1 | 0 | X | X |  | X | X |  |  |
| *Ischaemum* | 1 | 60 | 1 | 2.7 | 3 | 1 | X | X |  | X | X | X | X |
| *Sorghum* | 1 | 30 | 1 | 3 | 0 | 0 | X | X |  |  | X |  |  |
| *Saccharum* | 1 | 5 | 1 | 3.5 | 1 | 0 | X | X |  | X | X |  |  |
| *Imperata* | 1 | 8 | 0 | 2.7 | 3 | 1 | X |  |  | X | X | X | X |
| *Digitaria* | 1 | 220 | 0 | 2 | 2 | 1 | X |  |  |  | X | X |  |
| *Oplismenus* | 0 | 7 | 1 | 3 | 0 | 0 |  | X |  |  | X |  |  |
| *Poecilostachys* | 0 | 20 | 1 |  |  |  |  | X |  |  |  |  |  |
| *Cyrtococcum* | 0 | 12 | 1 | 3 | 0 | 0 |  | X |  |  | X |  |  |
| *Pseudechinolaena* | 0 | 6 | 1 | 3 | 0 | 0 |  | X |  |  | X |  |  |
| *Acroceras* | 0 | 15 | 1 | 4 | 2 | 0 | X | X | X | X | X |  |  |
| *Ottochloa* | 0 | 5 | 1 |  |  |  | X | X |  |  |  |  |  |
| *Alloteropsis* | 1 | 5 | 0 | 2.7 | 3 | 1 | X |  |  | X | X | X |  |
| *Echinochloa* | 1 | 30-40 | 0 | 3.5 | 1 | 0 | X |  | X | X | X |  |  |
| *Sacciolepis* | 0 | 30 | 0 | 4.5 | 1 | 0 | X |  | X | X |  |  |  |
| *Neurachne* | 1 | 6 | 0 | 1 | 0 | 1 | X |  |  |  |  | X |  |
| *Urochloa* | 1 | 3+120 | 1 | 2.7 | 3 | 1 | X | X |  | X | X | X |  |
| *Melinis* | 1 | 12 | 1 | 3.5 | 1 | 0 | X | X |  | X | X |  |  |
| *Stenotaphrum* | 1 | 7 | 0 | 3 | 0 | 1 | X |  |  |  | X |  | X |
| *Spinifex* | 1 | 4 | 0 | 1 | 0 | 1 | X |  |  |  |  | X | X |
| *Pennisetum* | 1 | 80 | 1 | 2.7 | 3 | 1 | X | X |  | X | X | X |  |
| *Cenchrus* | 1 | 22 | 1 | 2 | 2 | 1 | X | X |  |  | X | X | X |
| *Gynerium* | 0 | 1 | 0 | 4 | 0 | 0 |  |  |  | X |  |  |  |
| *Danthoniopsis* | 1 | 20 | 0 | 2 | 2 | 1 | X |  |  |  | X | X |  |
| *Tristachya* | 1 | 20 | 1 | 2.7 | 3 | 1 | X | X |  | X | X | X |  |
| *Loudetia* | 1 | 26 | 0 | 2.7 | 3 | 1 | X |  |  | X | X | X |  |
| *Cyperochloa* | 0 | 1 | 0 | 1 | 0 | 1 | X |  |  |  |  | X |  |
| *Centotheca* | 0 | 4 | 1 |  |  |  |  | X |  |  |  |  |  |
| *Thysanolaena* | 0 | 1 | 0 |  |  |  | X |  |  |  |  |  |  |
| *Chasmanthium* | 0 | 6 | 1 |  |  |  |  | X |  |  |  |  |  |
| *Zeugites* | 0 | 12 | 1 |  |  |  |  | X |  |  |  |  |  |
| *Orthoclada* | 0 | 2 | 1 |  |  |  |  | X |  |  |  |  |  |
| *Phragmites* | 0 | 3 |  | 4 | 0 | 0 |  |  |  | X |  |  |  |
| *Molinia* | 0 | 2-5 |  | 4 | 0 | 0 |  |  |  | X |  |  |  |
| *Arundo* | 0 | 3 |  | 3.5 | 1 | 0 |  |  |  | X | X |  |  |
| *Amphipogon* | 0 | 8 | 0 | 1 | 0 | 1 | X |  |  |  |  | X |  |
| *Micraira* | 0 | 13 |  |  |  |  |  |  |  |  |  |  |  |
| *Isachne* | 0 | 100 | 1 | 3.5 | 1 | 0 | X | X |  | X | X |  |  |
| *Eriachne* | 1 | 40 | 0 |  |  |  | X |  |  |  |  |  |  |
| *Karroochloa* | 0 | 4 | 0 | 3 | 0 | 0 | X |  |  |  | X |  |  |
| *Austrodanthonia* | 0 | 28 | 0 | 3 | 0 | 0 | X |  |  |  | X |  |  |
| *Cortaderia* | 0 | 24 | 0 | 2 | 2 | 1 | X |  |  |  | X | X |  |
| *Centropodia* | 1 | 4 | 0 | 1 | 0 | 1 | X |  |  |  |  | X |  |
| *Eragrostis* | 1 | 350 | 0 | 2 | 2 | 1 | X |  |  |  | X | X | X |
| *Uniola* | 1 | 2 | 0 |  |  | 1 | X |  |  |  |  |  | X |
| *Spartina* | 1 | 16 | 0 | 4.5 | 1 | 1 | X |  | X | X |  |  | X |
| *Calamovilfa* | 1 | 4 | 0 |  |  |  | X |  |  |  |  |  |  |
| *Crypsis* | 1 | 8 | 0 |  |  | 1 | X |  |  |  |  |  | X |
| *Sporobolus* | 1 | 160 |  | 2 | 2 | 1 |  |  |  |  | X | X | X |
| *Zoysia* | 1 | 10 | 0 | 1 | 0 | 1 | X |  |  |  |  | X | X |
| *Tragus* | 1 | 7 | 0 | 2 | 2 | 1 | X |  |  |  | X | X |  |
| *Distichlis* | 1 | 6 | 0 |  |  | 1 | X |  |  |  |  |  | X |
| *Tripogon* | 1 | 30 | 0 | 2.7 | 3 | 1 | X |  |  | X | X | X |  |
| *Dactyloctenium* | 1 | 13 | 0 | 2 | 2 | 1 | X |  |  |  | X | X | X |
| *Eleusine* | 1 | 9 | 0 | 2 | 2 | 1 | X |  |  |  | X | X |  |
| *Enteropogon* | 1 | 11 | 1 | 2 | 2 | 1 | X | X |  |  | X | X |  |
| *Chloris* | 1 | 55 | 0 | 2 | 2 | 1 | X |  |  |  | X | X |  |
| *Tetrapogon* | 1 | 5-6 | 1 | 3.5 | 1 | 0 | X | X |  | X | X |  |  |
| *Lepturus* | 1 | 8 | 0 | 1 | 0 | 1 | X |  |  |  |  | X | X |
| *Cynodon* | 1 | 10 | 0 | 2 | 2 | 1 | X |  |  |  | X | X | X |
| *Aristida* | 1 | 290 |  | 1 | 0 | 1 |  |  |  |  |  | X |  |
| *Sartidia* | 0 | 4 |  | 3 | 0 | 0 |  |  |  |  | X |  |  |
| *Stipagrostis* | 1 | 50 | 0 | 1 | 0 | 1 | X |  |  |  |  | X |  |
| *Nardus* | 0 | 1 | 0 | 1 | 0 | 1 | X |  |  |  |  | X |  |
| *Lygeum* | 0 | 1 | 0 | 1 | 0 | 1 | X |  |  |  |  | X |  |
| *Stipa* | 0 | 50 | 0 | 1 | 0 | 1 | X |  |  |  |  | X |  |
| *Ampelodesmos* | 0 | 1 |  | 1 | 0 | 1 |  |  |  |  |  | X |  |
| *Melica* | 0 | 80 | 1 | 2 | 2 | 1 | X | X |  |  | X | X |  |
| *Glyceria* | 0 | 40 |  | 4.5 | 1 | 0 |  |  | X | X |  |  |  |
| *Brachypodium* | 0 | 16 | 1 | 3 | 0 | 0 | X | X |  |  | X |  |  |
| *Elymus* | 0 | 150 |  | 2 | 2 | 1 |  |  |  |  | X | X |  |
| *Triticum* | 0 | 8 | 0 | 2 | 2 | 1 | X |  |  |  | X | X |  |
| *Hordeum* | 0 | 40 | 0 | 2 | 2 | 1 | X |  |  |  | X | X | X |
| *Bromus* | 0 | 150 | 1 | 2 | 2 | 1 | X | X |  |  | X | X |  |
| *Arrhenatherum* | 0 | 4 | 0 | 2 | 2 | 1 | X |  |  |  | X | X |  |
| *Avena* | 0 | 27 | 0 | 2 | 2 | 1 | X |  |  |  | X | X |  |
| *Agrostis* | 0 | 220 | 1 | 2.7 | 3 | 1 | X | X |  | X | X | X | X |
| *Aira* | 0 | 8 | 0 | 2 | 2 | 1 | X |  |  |  | X | X |  |
| *Dactylis* | 0 | 1 | 1 | 3 | 0 | 0 | X | X |  |  | X |  |  |
| *Holcus* | 0 | 9 | 1 | 3 | 0 | 0 | X | X |  |  | X |  |  |
| *Microlaena* | 0 | 10 | 1 | 3.5 | 1 | 0 | X | X |  | X | X |  |  |
| *Ehrharta* | 0 | 27 | 1 | 3.5 | 1 | 1 | X | X |  | X | X |  | X |
| *Zizania* | 0 | 3 |  | 4.5 | 1 | 0 |  |  | X | X |  |  |  |
| *Oryza* | 0 | 25 | 1 | 4.5 | 1 | 0 | X | X | X | X |  |  |  |
| *Streptogyna* | 0 | 2 | 1 |  |  |  |  | X |  |  |  |  |  |
| *Chusquea* | 0 | 100 |  | 4 | 0 | 0 |  |  |  | X |  |  |  |
| *Pariana* | 0 | 34 | 1 |  |  |  |  | X |  |  |  |  |  |
| *Olyra* | 0 | 23 | 1 | 3 | 0 | 0 |  | X |  |  | X |  |  |
| *Lithachne* | 0 | 4 | 1 |  |  |  |  | X |  |  |  |  |  |
| *Guaduella* | 0 | 8 | 1 |  |  |  |  | X |  |  |  |  |  |
| *Pharus* | 0 | 6 | 1 |  |  |  |  | X |  |  |  |  |  |
| *Anomochloa* | 0 | 1 | 1 |  |  |  |  | X |  |  |  |  |  |
| *Streptochaeta* | 0 | 3 | 1 |  |  |  |  | X |  |  |  |  |  |
