# Supplementary material for: Ecological selection pressures for C4 photosynthesis in the grasses
Source: Proc Biol Sci. 2009 Feb 25;276(1663):1753–60. doi: 10.1098/rspb.2008.1762 (PMC2674487; doi:10.1098/rspb.2008.1762)
Supplement: Table S2 — Fitted models [file rspb20081762s03.doc]

**Table S2.** Fitted Models. Summarised below are the fitted rates of transition between states and the 95% credible intervals determined from the posterior distribution of models. Also shown are the rate differences described in the text, which are used to test hypotheses concerning the differences between rate of transitions between states. The figures in brackets show the estimated mean when genera containing both C_3_ and C_4_ species are coded as being in the C_3_ rather than C_4_ state.

| 1. Shade vs. Open. C_4_ to C_3_ reversions allowed | | | |
| --- | --- | --- | --- |
| Rate | Mean Posterior | L95 | U95 |
| q12 | 0.67 (0.53) | 0.27 | 1.63 |
| q21 | 0.16 (0.22) | 0.00 | 0.79 |
| q13 | 0.24 (0.34) | 0.00 | 0.70 |
| q31 | 0.34 (0.22) | 0.00 | 0.82 |
| q24 | 0.00 (0.00) | 0.00 | 0.00 |
| q42 | 0.37 (0.34) | 0.00 | 1.12 |
| q34 | 0.43 (0.46) | 0.13 | 0.87 |
| q43 | 1.01 (0.78) | 0.30 | 3.02 |
| q13 - q24 | **0.23 (0.34)** | **0.01** | **0.70** |
| q12 - q34 | 0.23 (0.09) | -0.23 | 1.34 |
| q21 - q43 | **-0.85 (-0.56)** | **-2.99** | **-0.01** |
|  |  |  |  |
| 1. Shade vs. Open. No C_4_ to C_3_ reversions allowed. | | | |
| Rate | Mean Posterior | L95 | U95 |
| q12 | 0.66 (0.60) | 0.20 | 1.87 |
| q21 | 0.33 (0.27) | 0.04 | 0.92 |
| q13 | 0.61 (0.50) | 0.25 | 1.10 |
| q31 | - | - | - |
| q24 | 0.05 (0.05) | 0.00 | 0.19 |
| q42 | - | - | - |
| q34 | 1.13 (1.60) | 0.28 | 3.12 |
| q43 | 3.49 (4.70) | 1.02 | 9.36 |
| q13 - q24 | **0.56 (0.45)** | **0.14** | **1.07** |
| q12 - q34 | -0.47 (-1.00) | -2.69 | 1.28 |
| q21 - q43 | **-3.16 (-4.37)** | **-9.23** | **-0.51** |
|  |  |  |  |
| 1. Mesic vs. Xeric. C_4_ to C_3_ reversions allowed | | | |
| Rate | Mean Posterior | L95 | U95 |
| q12 | 0.42 (0.49) | 0.00 | 0.89 |
| q21 | 0.33 (0.33) | 0.00 | 0.93 |
| q13 | 0.11 (0.16) | 0.00 | 0.45 |
| q31 | 0.63 (0.54) | 0.17 | 1.22 |
| q24 | 0.04 (0.03) | 0.00 | 0.39 |
| q42 | 0.02 (0.02) | 0.00 | 0.21 |
| q34 | 0.85 (1.07) | 0.42 | 1.20 |
| q43 | 0.32 (0.33) | 0.03 | 0.85 |
| q13 - q24 | 0.06 (0.13) | -0.39 | 0.45 |
| q12 - q34 | **-0.42 (-0.58)** | **-1.70** | **-0.01** |
| q21 - q43 | 0.01 (0.00) | -0.78 | 0.77 |
|  |  |  |  |
| Mesic vs. Xeric. No C_4_ to C_3_ reversions allowed | | | |
| Rate | Mean Posterior | L95 | U95 |
| q12 | 0.24 (0.28) | 0.01 | 0.70 |
| q21 | 0.59 (0.47) | 0.21 | 1.16 |
| q13 | 0.45 (0.31) | 0.20 | 0.81 |
| q31 | - | - | - |
| q24 | 0.09 (0.00) | 0.02 | 0.30 |
| q42 | - | - | - |
| q34 | 3.16 (1.68) | 1.14 | 12.42 |
| q43 | 0.84 (0.15) | 0.06 | 4.94 |
| q13 - q24 | 0.36 (0.31) | -0.02 | 0.76 |
| q12 - q34 | **-2.92 (-1.46)** | **-8.63** | **-0.92** |
| q21 - q43 | -0.25 (0.32) | -4.47 | 0.91 |
