# Supplementary material for: Ecological selection pressures for C4 photosynthesis in the grasses
Source: Proc Biol Sci. 2009 Feb 25;276(1663):1753–60. doi: 10.1098/rspb.2008.1762 (PMC2674487; doi:10.1098/rspb.2008.1762)

Figure S1
Tree for analysis of shade vs. open states

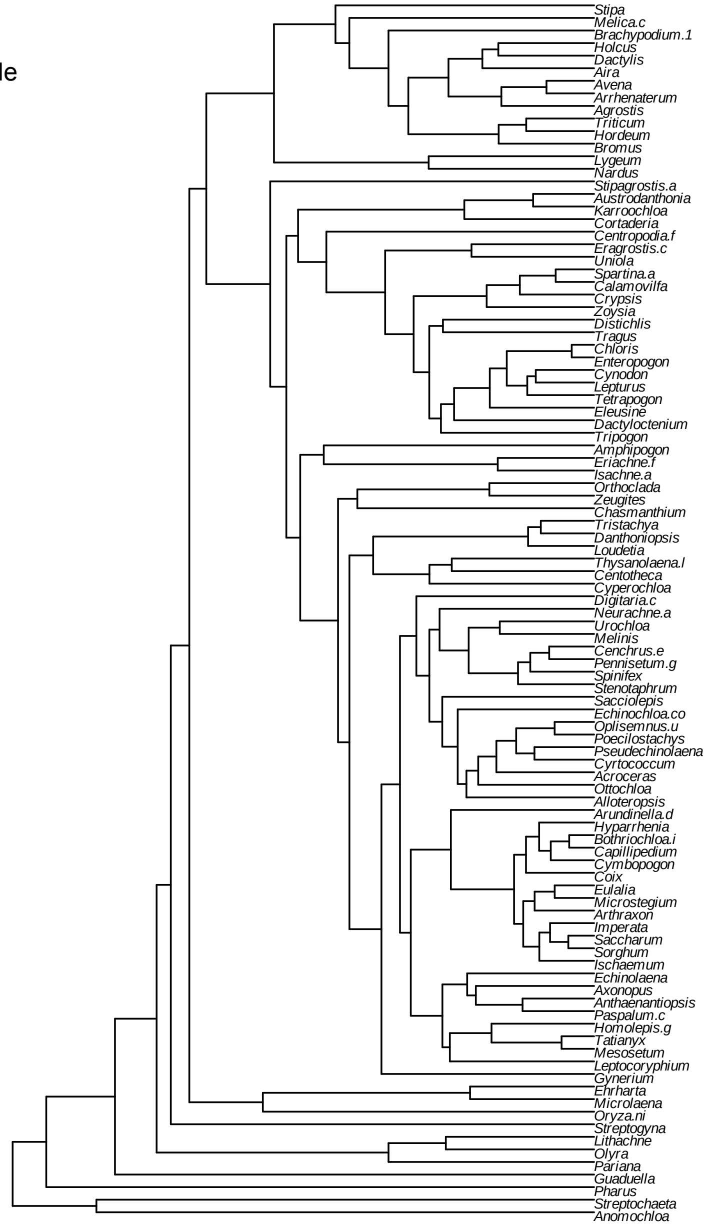

Figure S1
Tree for analysis of xeri vs. mesic states

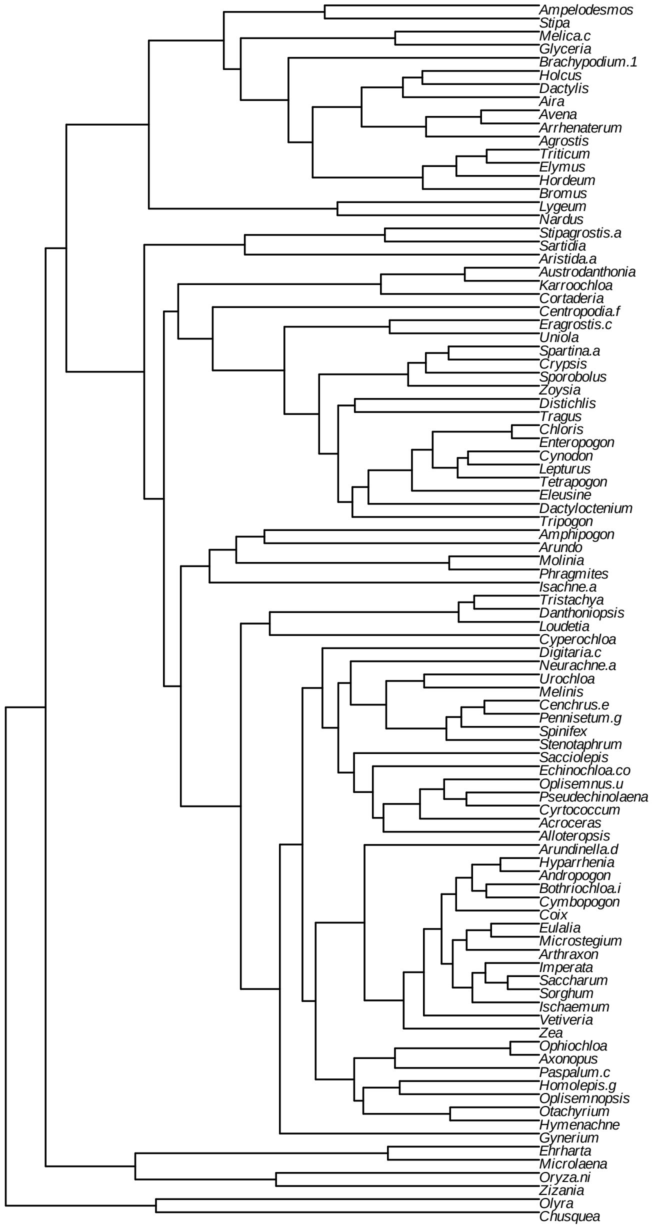

Supplement: Figure S1 — Key to the genera in figures 3 and 4 [file rspb20081762s04.pdf]
